# Polydatin attenuated neuropathic pain and motor dysfunction following spinal cord injury in rats by employing its anti-inflammatory and antioxidant effects

**DOI:** 10.3389/fphar.2024.1452989

**Published:** 2024-08-13

**Authors:** Faezeh Sadat Bagheri Bavandpouri, Atefeh Azizi, Fatemeh Abbaszadeh, Amir Kiani, Mohammad Hosein Farzaei, Ehsan Mohammadi-Noori, Sajad Fakhri, Javier Echeverría

**Affiliations:** ^1^ Student Research Committee, Kermanshah University of Medical Sciences, Kermanshah, Iran; ^2^ Neurobiology Research Center, Shahid Beheshti University of Medical Sciences, Tehran, Iran; ^3^ Pharmaceutical Sciences Research Center, Health Institute, Kermanshah University of Medical Sciences, Kermanshah, Iran; ^4^ Regenerative Medicine Research Center (RMRC), Kermanshah University of Medical Sciences, Kermanshah, Iran; ^5^ Departamento de Ciencias del Ambiente, Facultad de Química y Biología, Universidad de Santiago de Chile, Santiago, Chile

**Keywords:** spinal cord injury, polydatin, oxidative stress, inflammation, motor activity, neuropathic pain

## Abstract

**Background:**

Considering the complex pathological mechanisms behind spinal cord injury (SCI) and the adverse effects of present non-approved drugs against SCI, new studies are needed to introduce novel multi-target active ingredients with higher efficacy and lower side effects. Polydatin (PLD) is a naturally occurring stilbenoid glucoside recognized for its antioxidative and anti-inflammatory properties. This study aimed to assess the effects of PLD on sensory-motor function following SCI in rats.

**Methods:**

Following laminectomy and clip compression injury at the thoracic 8 (T8)-T9 level of the spinal cord, rats were randomly assigned to five groups: Sham, SCI, and three groups receiving different doses of PLD treatment (1, 2, and 3 mg/kg). Over 4 weeks, behavioral tests were done such as von Frey, acetone drop, hot plate, Basso-Beattie-Bresnahan, and inclined plane test. At the end of the study, changes in catalase and glutathione activity, nitrite level, activity of matrix metalloproteinase 2 (MMP2) and MMP9 as well as spinal tissue remyelination/neurogenesis, were evaluated.

**Results:**

The results revealed that PLD treatment significantly improved the behavioral performance of the animals starting from the first week after SCI. Additionally, PLD increased catalase, and glutathione levels, and MMP2 activity while reduced serum nitrite levels and MMP9. These positive effects were accompanied by a reduction in the size of the lesion and preservation of neuronal count.

**Conclusion:**

In conclusion, PLD showed neuroprotective effects in SCI rats by employing anti-inflammatory and antioxidant effects, through which improve sensory and motor function.

## 1 Introduction

Spinal cord injury (SCI) is a complex and multifaceted process that involves both primary and secondary damage to the spinal cord. Secondary injury refers to a series of biochemical and cellular processes that occur after the initial injury, and further worsening the harm to the spinal cord. Amongst those mechanisms, inflammation, oxidative stress, apoptosis, excitotoxicity, demyelination, and glial scar formation play critical roles (Anjum et al., 2020). In this regard, oxidative stress plays a significant role in causing neurological injuries. This occurs through the excessive production of reactive oxygen species (ROS), including free radicals and lipid peroxides. Several studies have demonstrated that levels of oxidative stress increase after SCI, leading to a decrease in the antioxidant defense potential, including activities of glutathione and catalase (Jia et al., 2012; Stewart et al., 2022a). Furthermore, SCI-induced oxidative stress also leads to the modification of signaling pathways. Studies have revealed that an increase in inducible nitric oxide synthase (iNOS), the crucial enzyme responsible for the synthesis of nitric oxide (NO), increased following SCI (Conti et al., 2007). NO has been found to inhibit the expression of matrix metalloproteinase-2 (MMP2) (Chen and Wang, 2004). Hence, employing pharmacological intervention to protect against these effects could be a promising therapeutic strategy. Researchers have increasingly focused on exploring the pharmacological effects of natural products on various diseases due to their wide range of beneficial activities and minimal side effects.

Polydatin (PLD), 3,4,5-Trihydroxystilbene-3-beta-monoglucoside or piceid, is a naturally occurring stilbenoid glucoside and glycosided form of resveratrol. PLD is the main bioactive ingredient extracted from the roots of the *Reynoutria japonica* Houtt (Polygonaceae), bark of *Picea sitchensis* (Bong.) Carrière (Pinaceae). In addition, PLD also can be found in significant amounts in common botanical sources, as grapes, hop cones, peanuts, and red wines. PLD is used as chemomarker to characterize the quality of *Reynoutria japonica* in the Pharmacopoeia of the People’s Republic of China where is traditionally used for treatment of hyperlipemia, inflammation, infection and cancer. PLD exhibits a range of beneficial properties, including anti-inflammatory, immunoregulatory, antioxidant, and anti-tumor activities (Karami et al., 2022). In a comparative study, it was found that PLD has superior and higher activity in inhibiting hydroxyl radicals than resveratrol (Wang et al., 2015). As a potent stilbenoid polyphenol, PLD has the ability to modulate crucial signaling pathways associated with inflammation, oxidative stress, and apoptosis, thereby exhibiting potential biological activities (Fakhri et al., 2021a). Additionally, PLD exhibited greater antioxidant (Şöhretoğlu et al., 2018) and anti-inflammatory (Lanzilli et al., 2012) effects compared to its glycoside derivative, resveratrol. As well, PLD possesses superior biodistribution and antioxidant activity. Moreover, PLD demonstrates higher intestinal absorption due to the presence of its sugar groups (Wang et al., 2015), distinguishing it from resveratrol. Overall, considering the critical role of inflammation, oxidative stress and apoptosis behind neurodegeneration and the aforementioned multi-targeting potential of PD with more bioavailability in the regulation of multiple dysregulated pathways, in the present study, we examined the neuroprotective benefits of intrathecal (i.t.) administration of PLD after SCI.

## 2 Materials and methods

### 2.1 Chemical and reagents

Polydatin and cefazolin was purchased from Sigma–Aldrich (Sigma Chemical Co., St. Louis, MO, United States) and Exir company (Exir company, Iran, i.p.), respectively. All the other chemicals and reagents were of analytical reagent grade purchased from commercial sources.

### 2.2 Experimental animals

In total, 35 male Wistar rats, aged 8–9 weeks, were obtained and kept in controlled conditions with equal light/darkness (12 h) at the animal house of Kermanshah University of Medical Sciences (temperature 24°C ± 2°C, with fresh water and food *ad libitum*, 10 days prior to the beginning of study). The study was conducted under the guidelines of this university’s animal care committee (IR.KUMS.REC. 1400.388, **Supplementary material**). The male rats were allocated into the following groups: Sham, SCI groups that received distilled water as a solvent, and three treatment groups that received doses of 1, 2, and 3 mg/kg of PLD, respectively. Male rats included in ours research, since they have more consistent hormonal levels in comparison to females, which could be influenced by their estrous cycle specially in rat behavior (Zeng et al., 2023).

### 2.3 Spinal cord injury

At the start of surgery, rats were anesthetized with ketamine and xylazine (80/10 mg/kg, intraperitoneal (i.p.). The thoracic vertebrae (T8-T9) were removed using a micro rongeur (Fine Science Tools, United States. To create a compression model of SCI, rats in the SCI and PLD groups were subjected to a force of 90 g for 1 min using an aneurysm clip (Aesculap, Tuttlingen, Germany). The muscles and skin were sutured after the surgery. 30 min after SCI, rats received 10 μL of doses of 1, 2, and 3 mg/kg PLD or distilled water intrathecally. 40 mg/kg of cefazolin (i.p.) (Exir company, Iran, i.p.) and 2 mL of normal saline (subcutaneous) and manual bladder massage were performed as post-surgery care until the return of normal bladder function (Fakhri et al., 2018; Fakhri et al., 2019).

### 2.4 Behavioral test

Behavioral tests were conducted on all animals before surgery (day 0) and weekly after surgery on days 7, 14, 21, and 28.

#### 2.4.1 Cold allodynia

To assess cold sensitivity in rats, a few drops of acetone (100 μL) were sprayed onto the soles of the animals from a distance of 200 mm. The response of the hind paw was observed and scored as follows: no paw withdrawal (scored as 0), startle response without paw withdrawal (scored as 1), partial withdrawal of the paw, 5–30 s (scored as 2), prolonged withdrawal of the paw, >30 s (scored as 3), and licking and jumping (scored as 4) (Kauppila, 2000; Fakhri et al., 2018).

#### 2.4.2 Heat hyperalgesia

To measure hyperalgesia, each animal was placed in a plexiglass chamber on a hot plate device (Harvard device, United States, 50°C ± 2°C). The delay time for paw withdrawal, indicated by licking or jumping, was recorded as paw withdrawal latency (PWL). To prevent tissue damage, a cut-off time of 1 min was considered (Fakhri et al., 2022b).

#### 2.4.3 Mechanical allodynia

The assessment of mechanical allodynia was carried out by using von Frey filaments. Both lateral plantar areas of the paw were tested with a series of von Frey strings (10, 15, 26, 60, and 100 g). Each strand was applied five times. 3 positive answers (leg dragging) out of 5 tests were considered as the allodynia threshold (Fakhri et al., 2019).

#### 2.4.4 Inclined plane test

The inclined plane test was used to evaluate the motor function of rats after SCI. In this experiment, the rats were placed on an inclined plane with a variable angle between 0 and 60. The maximum angle at which the animal kept its balance for at least 5 s was taken as the response (Fakhri et al., 2018).

#### 2.4.5 Basso-Beattie-Bresnahan (BBB) scores

The Basso-Beattie-Bresnahan (BBB) score test was another locomotor test used to evaluate the motor function of rats. The test involved observing the rat’s hind limb movements for 4 min and assigning a score based on the degree of movement and coordination. The score ranges from 0 (no movement) to 21 (normal movement). The average score of both paws was considered as the final response (Fakhri et al., 2021b).

### 2.5 Biochemical test

At the end of the behavioral evaluation (day 28), the rats were sacrificed and their serum samples were used for biochemical studies.

#### 2.5.1 Nitrite assay

Nitric oxide levels in serum samples are assessed by the Griess assay. Therefore, this test provides an indirect indication of NO levels. Griess reagent was prepared from a solution of sulfonamide (dissolved in 5% phosphoric acid) and naphthyl ethylenediamine dihydrochloride (NEDD). In each of the wells of the plate, a mixture of serum sample and sulfonamide solution was poured. After minutes, 50 mL of NEDD solution was added. After a 5-minute incubation, the optical density (OD) was measured at 540 nm. The standard curve was plotted simultaneously using different concentrations of sodium nitrite (Sun et al., 2003).

#### 2.5.2 Glutathione/catalase assay

Changes in glutathione and catalase levels were evaluated to determine antioxidant levels. For the evaluation of glutathione, we employed Ellman’s reagent. To perform the test, we mixed 50 μL of phosphate-buffered saline (PBS), 40 μL of the serum sample, and 100 μL of 5,5'-dithio-bis(2-nitrobenzoic acid) (DTNB). This mixture was incubated for 10 min. Finally, we measured the optical density (OD) of each well at 412 nm using a plate reader (Eyer and Podhradský, 1986).

The catalase activity was measured with the Aebi method (Aebi, 1984). First, the combination of serum samples and hydrogen peroxide (65 mM) was incubated in the plate well for 4 min at 25°C. In order to stop the reaction, 100 µL ammonium molybdate (32.4 mM) was then added. Finally, an optical density reader was used to measure OD at 405 nm (Fakhri et al., 2022b).

The percentage difference in concentration between the sham group and other groups can be calculated as: (C_sham − C_samp)/C_sham) × 100.

### 2.6 The activity of MMP2 and MMP9

To assess the activity of MMP-2 and MMP-9 gelatin zymography was performed. Following doing Bradford protein assay, serum protein was loaded at 100 μg per sample and electrophoresis was used with a voltage of 150 V. Gels were washed in a renaturation buffer (Triton™ X-100 in Tris-HCl, pH 7.5) on the shaker. In following, the gels were incubated for 18 h at 37°C in an incubation buffer consisting of NaN_3_, NaCl, and CaCl_2_ in Tris-HCl. Then, gels were stained with Coomassie blue and destained in an acetic acid and methanol solution. Image j software was used to evaluate the bands area and intensity (Fakhri et al., 2021b).

### 2.7 Histological analysis

Rats were perfused transcardially with approximately 250 mL of PBS (pH 7.3) and 300 mL of 4% paraformaldehyde (PFA) on days 7, 21, and 28. The tissue from the injury site of the spinal cord was collected and prepared using a tissue processor. Subsequently, it was embedded in paraffin. The paraffinized tissue was then cut into sequential sections and stained with hematoxylin and eosin (H&E). These stained sections were observed under a light microscope at both 10× and 40× magnification. To analyze the injury site, as well as the number of neurons in the dorsal and ventral horns, Image J software developed by National Institutes of Health (NIH) was utilized for quantification (Fakhri et al., 2018; Fakhri et al., 2019; Fakhri et al.,2021b).

### 2.8 Statistical analysis

The data has been presented in the form of mean ± standard error of the mean (SEM), and it has been analyzed using GraphPad Prism software (version 8.4.3). One-way and two-way analysis of variance (ANOVA) have been applied, followed by Tukey’s and Bonferroni’s *post hoc* analyses. A statistically significant difference has been considered at *p* < 0.05.

## 3 Results

### 3.1 Behavioral result

#### 3.1.1 Cold allodynia

**FIGURE 1:**
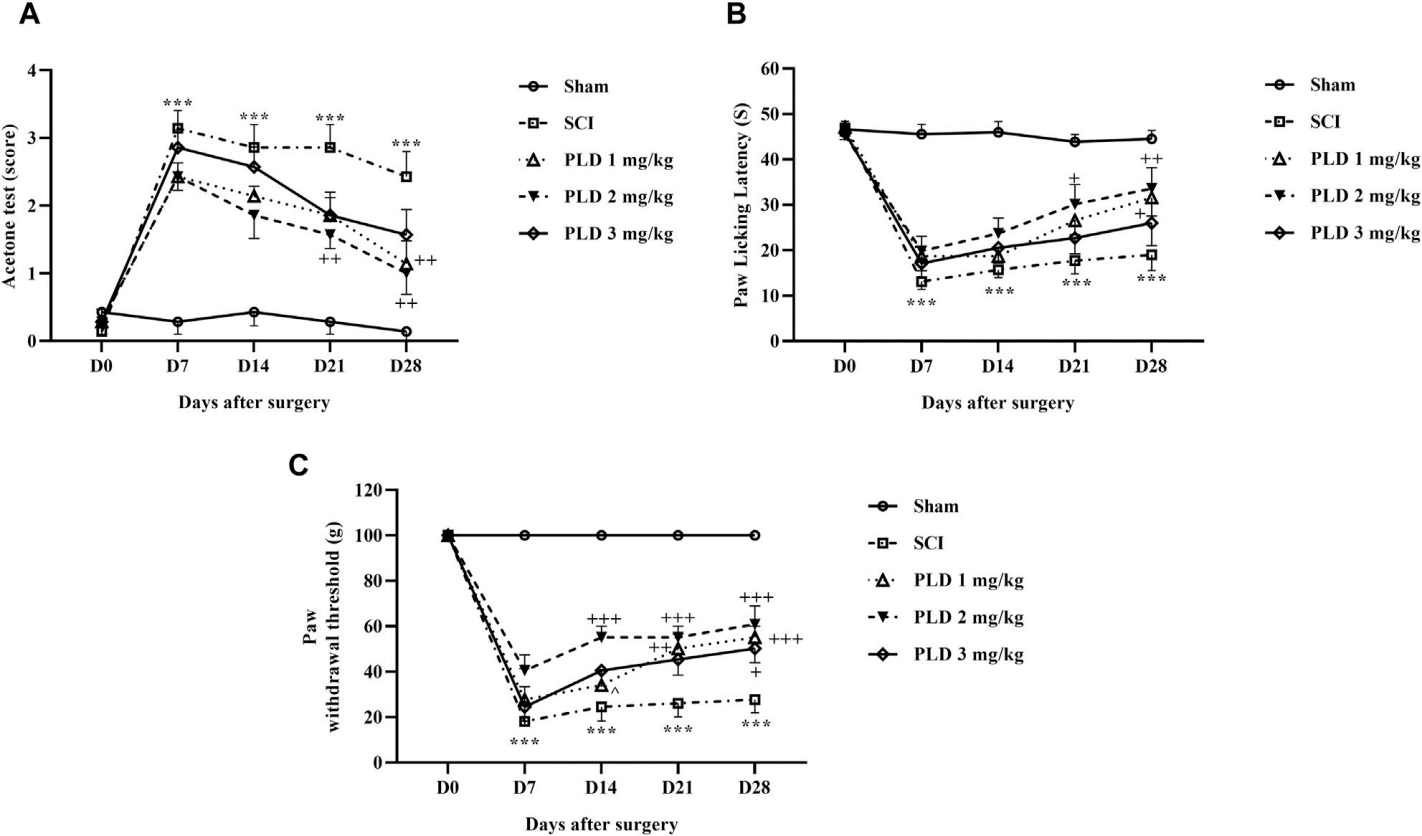
The impact of PLD on behaviors related to pain after SCI. The paw response threshold to cold **(A)**, thermal **(B)**, and mechanical **(C)** stimuli were measured and the data is presented as the mean ± SEM (*n* = 7). The statistical analysis was done using a two-way ANOVA. ^***^ *p* < 0.001 vs. sham group; ^+^ *p* < 0.05, ^++^ *p* < 0.01, ^+++^ *p* < 0.001 vs. SCI group; ^^^ *p* < 0.05 vs. PLD 2 mg/kg group.

Cold pain response threshold data were subjected to two-way ANOVA analysis. It was found that during the 4-week follow-up, rats in the sham group continued to have normal sensitivity to cold on all the investigated days. However, rats in the SCI group showed significant hypersensitivity to cold compared to the sham group (*p* < 0.001). Furthermore, animals treated with different doses of PLD compared to the SCI group had a higher response threshold to cold stimulation and the best results were obtained after 2 mg/kg treatment (*p* < 0.01) (Figure 1A).

#### 3.1.2 Heat hyperalgesia

The results of the threshold for responding to a thermal pain stimulus are displayed in the Figure 1B. The findings indicate that the paw-licking latency was consistently similar on all test days for the control group (sham). However, the animals in the SCI group became highly sensitive to the thermal pain stimulus. Administration of PLD increased the response threshold to heat stimulus in injured animals, and intrathecal injection of 2 mg/kg PLD was more effective than the other two doses (*p* < 0.01). (Figure 1B).

#### 3.1.3 Mechanical allodynia

The paw withdrawal responses to mechanical stimulation were almost the same in the sham group after 4 weeks. However, SCI caused a significant and sustained reduction in the paw withdrawal threshold (*p* < 0.001), indicating the presence of mechanical allodynia. Importantly, treatment with PLD, particularly at a dose of 2 mg/kg, effectively reduced the symptoms induced by SCI from day 7 onwards (*p* < 0.001) (Figure 1C).

#### 3.1.4 Motor activity

**FIGURE 2:**
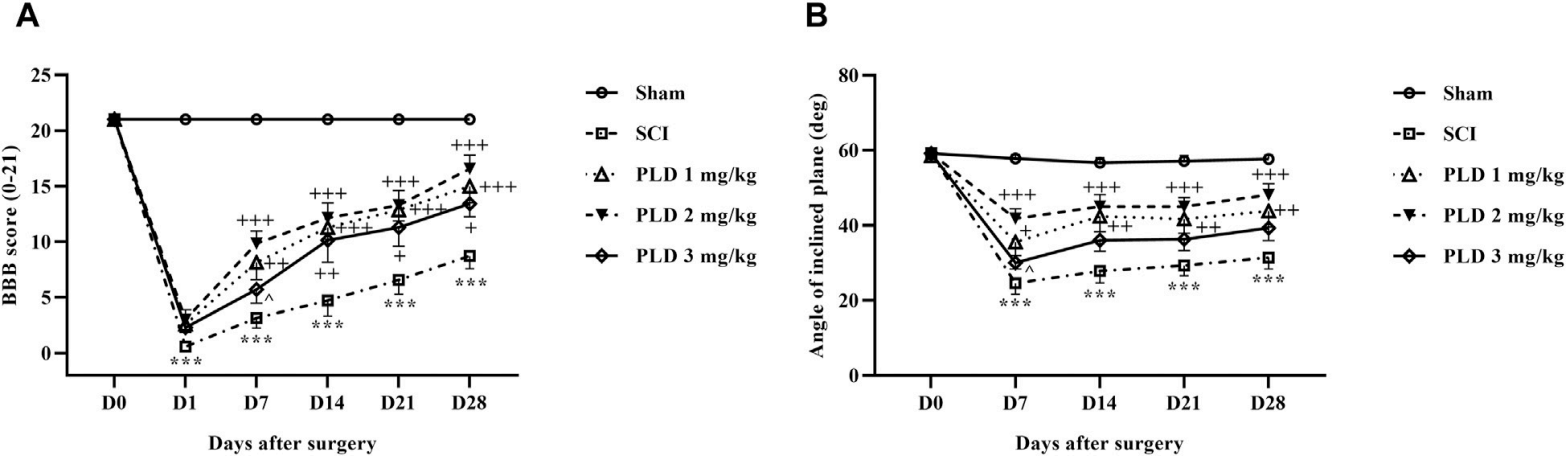
The impact of PLD on motor ability after SCI. The BBB score **(A)**, and inclined-plane **(B)** test were measured and the data is presented as the mean ± SEM (*n* = 7). The statistical analysis was done using a two-way ANOVA. ^***^ *p* < 0.001 vs. sham group; ^+^ *p* < 0.05, ^++^ *p* < 0.01, ^+++^ *p* < 0.001 vs. SCI group; ^^^ *p* < 0.05 vs. PLD 2 mg/kg group.

The motor behavior of rats following SCI was evaluated using the BBB scale and inclined plane test. The sham group had a stable score of 21, indicating that the laminectomy surgical did not cause any functional impairments. In contrast, the SCI rats showed significantly lower scores of 0 and 2 on the first day after the injury, and this reduction in motor performance persisted until day 28 post-SCI (*p* < 0.001). Interestingly, the administration of various doses of PLD effectively improved the motor function of the SCI rats, starting from day 1, when compared to the SCI group (*p* < 0.001) (Figure 2A).

The sham group maintained a consistent ability to stand and balance on the ramp over 4 weeks, averaging at approximately 60°C. However, the group with SCI experienced a significant decline in average balance angle, compared to the sham group (*p* < 0.001). Rats treated with various doses of PLD, especially the dose of 2 mg/kg showed substantial improvement from the first week (*p* < 0.001) (Figure 2B).

### 3.2 Biochemical result

#### 3.2.1 Nitrite assay

**FIGURE 3:**
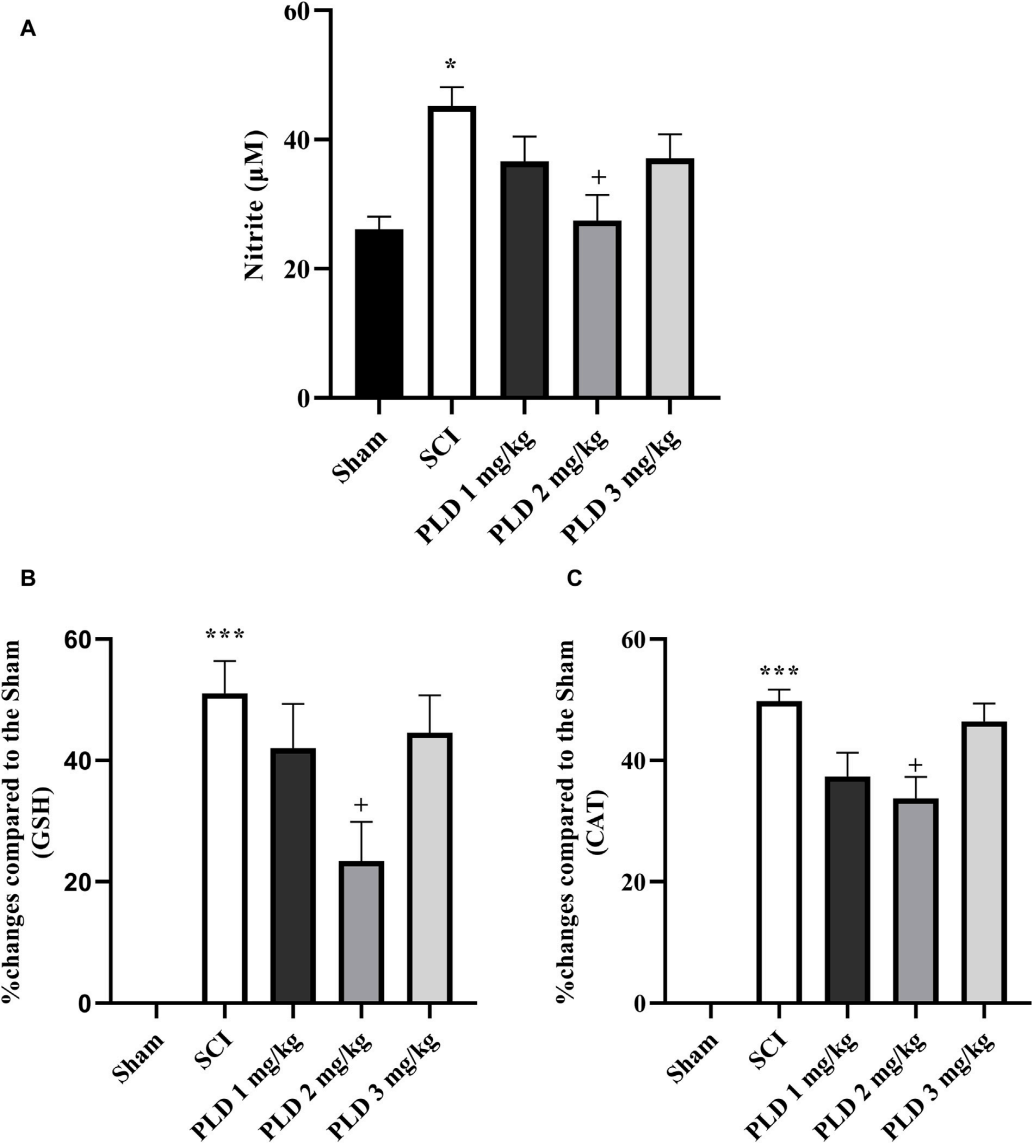
Effect of PLD on changes of oxidative stress. The level of nitrite **(A)**, and percentage of activity changes of glutathione **(B)** and catalase **(C)** were measured and the data is presented as the mean ± SEM (*n* = 3). The statistical analysis was done using a one-way ANOVA. ^*^ *p* < 0.05 and ^***^ *p* < 0.001 vs. sham group; ^+^ *p* < 0.05 vs. SCI group.

In comparison to the sham group, the SCI group showed a significant increase in serum nitrite levels (*p* < 0.05). While treatment with PLD 2 mg/kg resulted in a decrease in the serum nitrite level (*p* < 0.05) in comparison to the SCI group (*p* < 0.05). Although the other doses of PLD (1 mg/g and 3 mg/kg) also decreased serum levels of nitrite, it was not significant (Figure 3A)

#### 3.2.2 Catalase and glutathione assay

According to the results, the SCI group showed a significant reduction in the serum levels of catalase and glutathione compared to the sham group (*p* < 0.001). However, treatment with PLD, particularly at a dose of 2 mg/kg, was able to effectively compensate for this decrease (*p* < 0.01). Figures 3B, C presents that the administration of PLD 2 mg/kg decreased the differences between the group and sham group (see formula in Section 2.5.2). Other doses of PLD (1 and 3 mg/kg) also decreased the differences between groups and sham group, however those was not significant (Figures 3B, C).

### 3.3 MMP2 and MMP9 activity

**FIGURE 4:**
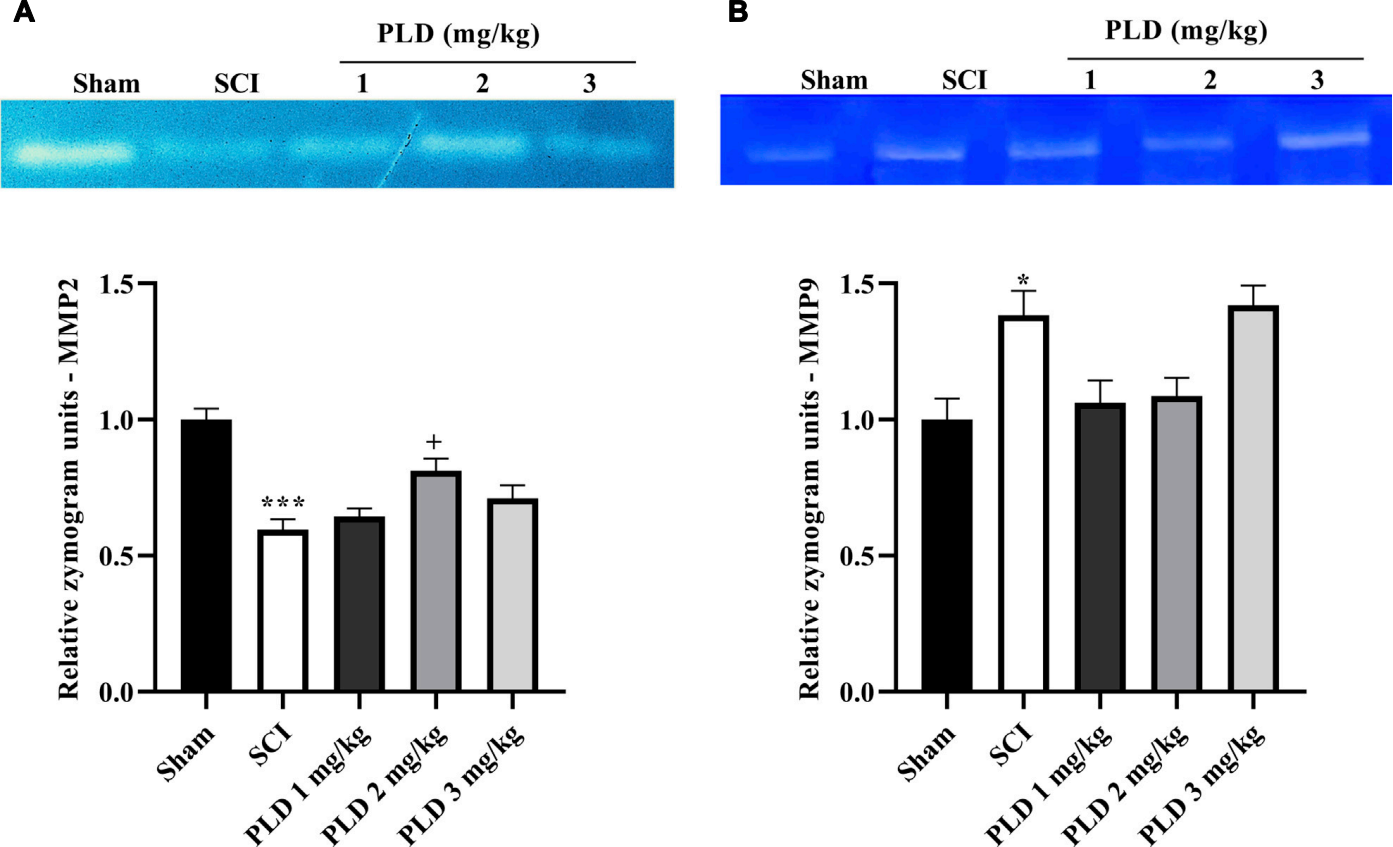
The impact of PLD on MMP-2 **(A)** and MMP-9 **(B)** activity after compression SCI. Data presented as mean ± SEM. ^*^ *p* < 0.05, ^***^ *p* < 0.001 vs. sham, ^+^ *p* < 0.05 vs. SCI group.

In day 28, the SCI group showed an elevation in MMP-9 activity while a reduction in MMP-2 in comparison to the sham group (*p* < 0.001). However, with PLD treatment, those changes reversed. The results showed that PLD 2 mg/kg potentially elevated anti-inflammatory MMP2 (*p* < 0.05). However, PLD made a non-significant reduction in the activity of MMP9 (Figures 4A, B).

### 3.4 Histological analysis

**FIGURE 5:**
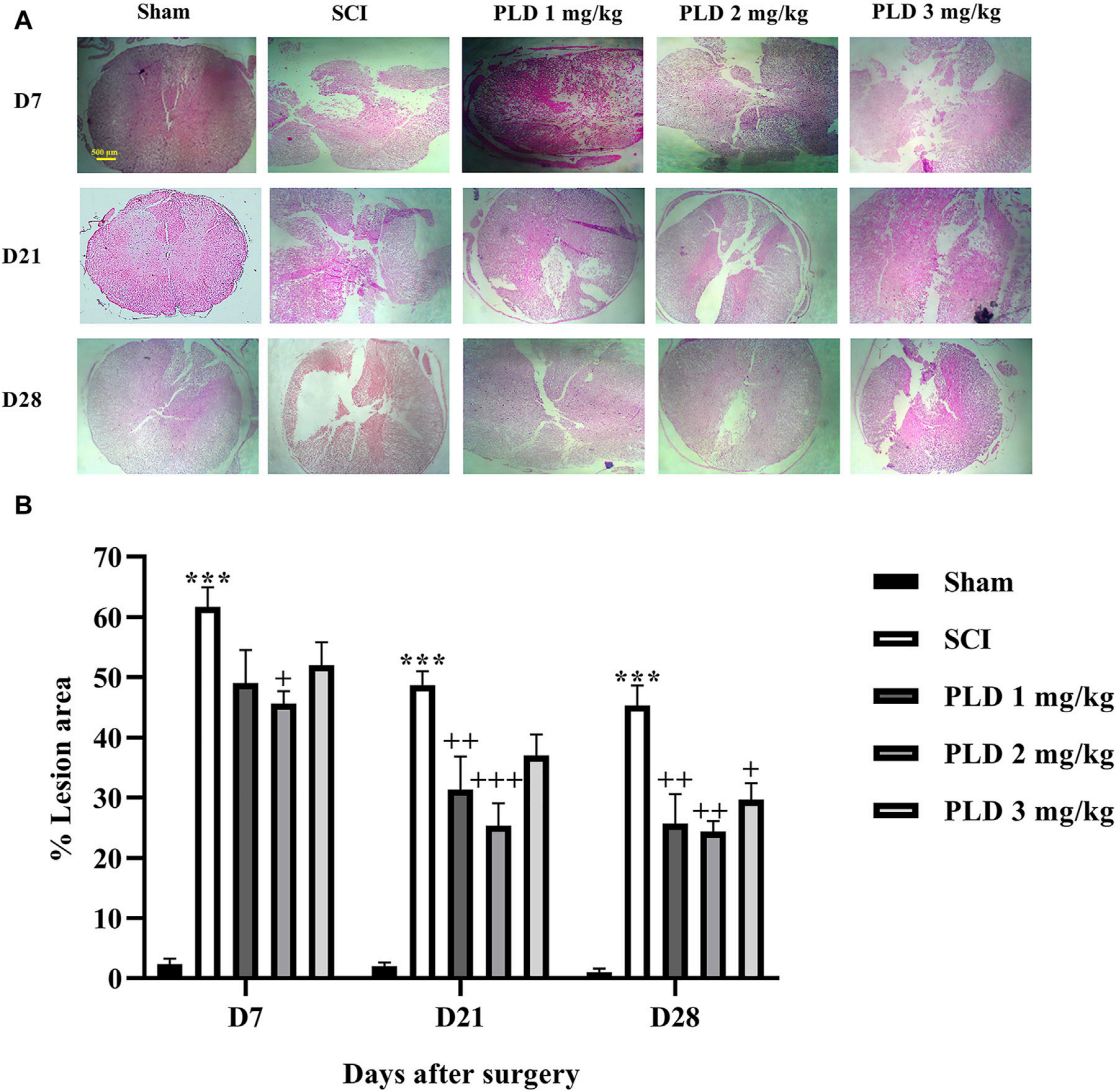
The impact of PLD on the lesion size after SCI **(A)** and associate analysis **(B)**. The data is presented as the mean ± SEM (*n* = 3). The statistical analysis was done using a two-way ANOVA. ^***^ *p* < 0.001 vs. sham group;+*p* < 0.05, ^++^ *p* < 0.01, ^+++^ *p* < 0.001 vs. SCI group; ^^^ *p* < 0.05 vs. PLD 2 mg/kg group. The scale bar (yellow line) shows 500 μm.

**FIGURE 6:**
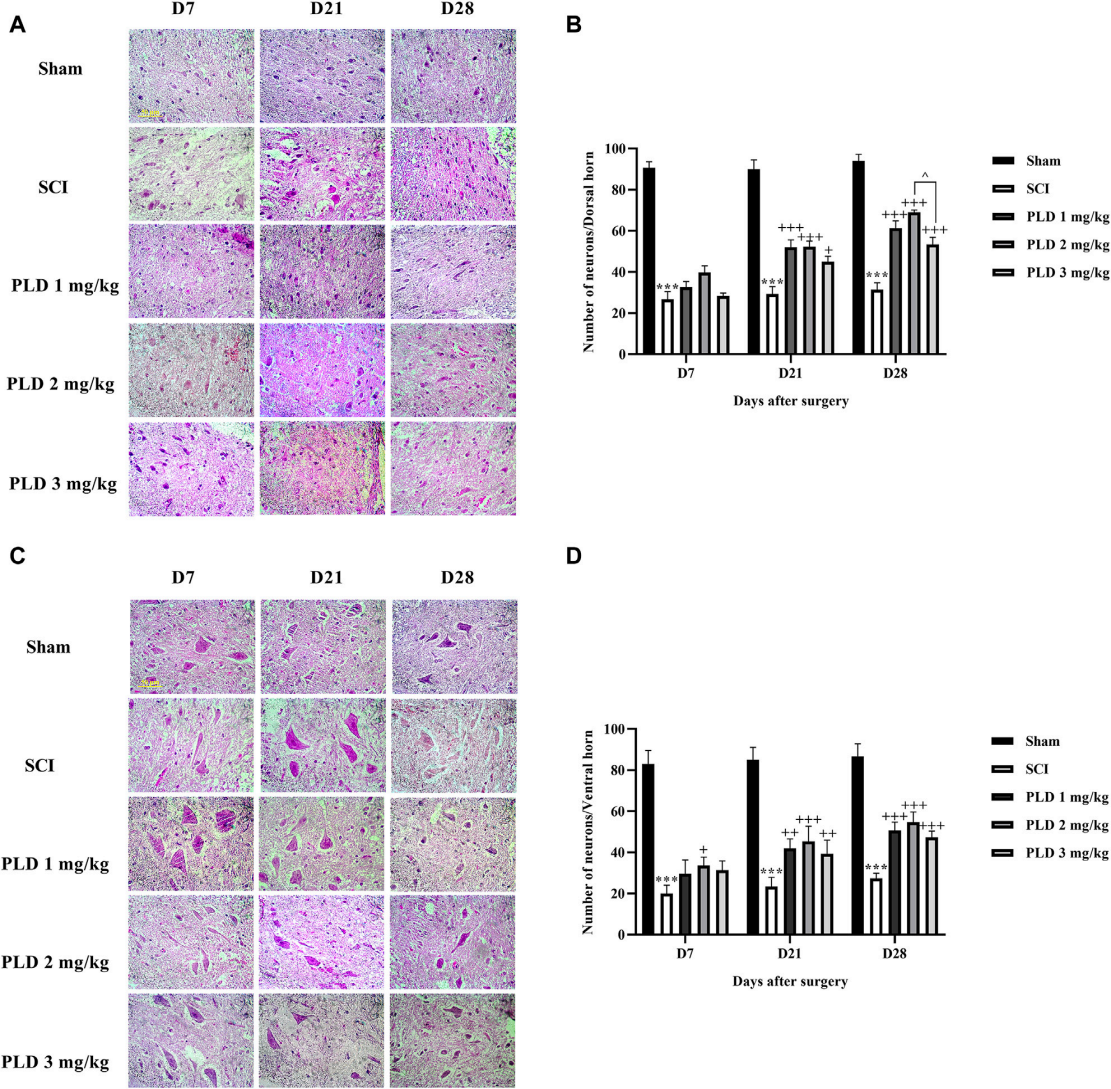
The impact of PLD on the number of neurons in both horns of the spinal cord after SCI. The dorsal **(A, B)** and ventral **(C, D)** horns of the spinal cord were measured and the data is presented as the mean ± SEM (*n* = 3). The statistical analysis was done using a two-way ANOVA. ^***^ *p* < 0.001 vs. sham group; ^+^ *p* < 0.05, ^++^ *p* < 0.01, ^+++^ *p* < 0.001 vs. SCI group; ^^^ *p* < 0.05 vs. PLD 2 mg/kg group. The scale bar (yellow line) shows 50 μm.

The histopathological analysis revealed that in the SCI group, there was a notable increase in the lesion size (Figures 5A, B) and a significant decrease in the number of neurons in both the dorsal (Figures 6A, B) and ventral (Figures 6C, D) horns of the spinal cord, as compared to the sham group (*p* < 0.001). On the contrary, in the groups treated with PLD, there was a remarkable decrease in the size of the lesion during 1 month. This reduction in the size of the lesion was accompanied by an increase in the number of neurons in both horns of the spinal cord (*p* < 0.001).

## 4 Discussion

The results of this study showed that treatment with PLD can significantly improve sensory and motor function and increase the number of dorsal-ventral spinal cord neurons and lesion size. In addition, PLD increased the level of catalase and glutathione, as well as MMP2 activity while reducing nitrite levels and MMP9 activity to protect the spinal cord tissue from secondary damages caused by oxidative stress.

SCI can be divided into two primary components: mechanical damage from temporary spinal cord compression and the development of early post-injury lesions. Subsequently, a series of additional damages can worsen nerve dysfunction and result in irreversible harm (Hu et al., 2023). The inflammatory response and apoptosis in the affected tissue are triggered by oxidative stress (Chandra et al., 2000; Zhang et al., 2012). Actually, after SCI, oxidative stress has been linked to the development of neuropathic pain through its ability to promote inflammation, nerve damage, and increased sensitivity to pain. Reactive oxygen and nitrogen species (ROS and RNS) can cause harm to DNA, lipids, and proteins, resulting in inflammation and disruption of cellular and tissue function (Hassler et al., 2014; Carrasco et al., 2018).

Research has been conducted on PLD to explore its potential health advantages, such as its antioxidant and anti-inflammatory properties (Chen et al., 2021). PLD is a resveratrol derivative (glucoside of resveratrol) with improved bioavailability. PLD has been compared to resveratrol in several studies, and it has been found to have a higher activity of scavenging hydroxyl radicals than resveratrol (Wang et al., 2015). Research has shown that PLD can modulate nitric oxide levels, and improve catalase and glutathione activity, thereby contributing to its antioxidant and anti-inflammatory effects (Karami et al., 2022). Our findings also confirmed these results (Increasing the level of catalase and glutathione and decreasing the level of nitrite).

Arriving at the most effective dose is a critical key point in combating neurodegeneration; though, this process would be challenging considering interindividual variation affected by age, gender, genetics, exercise, diet, and health status. Hormesis is a biphasic dose-response relationship known by high-dose inhibition and low-dose stimulation of biological responses, characterized by either graphical J/U-shaped or an inverted U-shaped dose-response curve. Hormetic responses are affected by several stimuli, including chemical exposures, dietary restriction, thermal/light/electricity extremes, hypoxia, ionizing radiation, and physical stress (Fakhri et al., 2022a). Based on the hormesis dose-response relationship, our study showed a better neuroprotective effect for PLD 2 mg/kg than 1 and 3 mg/kg in the regulation of motor dysfunction and neuropathic pain by combating inflammation and oxidative stress, as well as improving neuronal survival and spinal tissue repair.

PLD has been found to inhibit the production of pro-inflammatory molecules, such as cytokines and prostaglandins, which are involved in the signaling of pain. By reducing inflammation, PLD may help to alleviate pain associated with inflammatory conditions, such as arthritis or muscle strain (Karami et al., 2022; Zhang et al., 2024). Additionally, PLD has been shown to have antioxidant properties, which can help to reduce oxidative stress and damage in the body (Chen et al., 2015). Oxidative stress has been linked to the development and maintenance of chronic pain conditions, and by neutralizing free radicals and reducing oxidative damage, PLD may help to alleviate pain (Luo et al., 2022). Xi et al. reported that PLD attenuated vincristine-induced neuropathic pain in rats by suppressing the activation of inflammatory factors and inhibiting oxidative stress (Xi et al., 2022). Another study demonstrated that PLD attenuated experimental diabetic neuropathy in rats by suppressing the activation of inflammatory factors and inhibiting oxidative stress (Bheereddy et al., 2021). On the other hand, the reduction of pain was linked to improved motor performance. Lv et al. found that when rats were given intraperitoneal PLD, their locomotor activity improved. This has been attributed to the suppression of oxidative stress and apoptosis through the nuclear factor erythroid 2-related factor 2 (Nrf2)/heme oxygenase 1 (HO-1) pathway (Lv et al., 2019b). The mediators of oxidative stress has been found to inhibit the expression of MMP2 and elevate the activity of MMP9 (Chen and Wang, 2004). In our study, SCI made an increase in nitrite level, while decreased in catalase and glutathione, thereby increased the activity of MMP9 and decreased those of MMP2. These biochemical and zymography results made increased lesion size and suppressed numbers of sensory/motor neurons in rats. In terms of histology, studies have shown that PLD can promote axonal regeneration, improve histological damage, inhibit apoptosis levels, and reduce tissue damage after SCI (Lv et al., 2019a; Zhan et al., 2022). In the current study, PLD was also able to reduce the size of the lesion after SCI and preserve the dorsal and ventral horn neurons.

**FIGURE 7:**
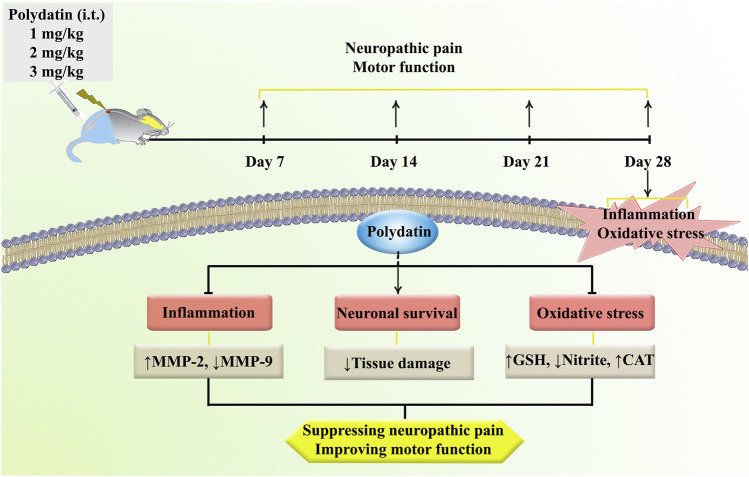
A description of the research methodologies and the regulatory role of PLD in the treatment of neuropathic pain and motor dysfunction via anti-inflammatory and antioxidant mechanisms.

We also found that SCI decreased the survival rate of sensory/motor neurons in the ventral/dorsal horns of spinal cord, respectively. These changes were reverted following PLD administration, which were in line with the behavioral results. Consistent with these results, we also found that PLD increased the response to cold, heat, and mechanical stimuli as well as maintaining limb movement following SCI by regulating inflammation/oxidative stress and histological protection (Figure 7).

Amongst SCI experimental models regarding examining the therapeutic effect and neuroprotective study following SCI, the compression model is a better experimental choice. Moreover, compression SCI is useful to evaluate the minimal loss of neurons after SCI. Compression SCI is also beneficial to study the secondary mechanisms and cell transplantation therapies (Sun et al., 2017). On the other hand, some experimental models of SCI possess minor limitations, however we used compression SCI which is more suitable for translational research and similar to those in SCI patients (Cregg et al., 2014). Regarding possible experimental limitations in SCI research protocols, pre-clinical SCI models commonly use young adult animals, while clinical models engages human subjects with wide age ranges (Stewart et al., 2022b). Other limitations include the inability to directly apply long-distance axon regeneration, which is required in humans to treat spinal injuries, to animal models. Larger volumes of reinnervation-needed gray matter in humans makes slower recovery and reduces spontaneous recovery in human after SCI (Kjell and Olson, 2016). Another important limitation of the compression method is that after SCI some neural tracts may not be well damaged leading to undesired results in regeneration research. Furthermore, about 4–6 weeks post-injury, tissue sparing may increase, presenting that the late subacute and early intermediate stages of the secondary injury are related to limited neuroanatomical recovery (Ahmed et al., 2019).

## 5 Conclusion

Our findings suggest that PLD can improve sensory-motor dysfunction caused by SCI in rats. This is due to the effective antioxidative properties of PLD, which reduced nitrite levels, enhanced catalase, and glutathione activity, ultimately leading to a reduction in histological damages. These results indicated that PLD has therapeutic potential for individuals experiencing SCI-related dysfunction.

## Data Availability

The raw data supporting the conclusions of this article will be made available by the corresponding author upon request.

## Abbreviations

ANOVA, analysis of variance; BBB, Basso-Beattie-Bresnahan; DTNB, 5,5'-dithio-bis(2 nitrobenzoic acid); H&E, hematoxylin and eosin; HO-1, heme oxygenase 1; iNOS, Inducible nitric oxide synthase; ip, intraperitoneal; MMP2, matrix metalloproteinase-2; MMP9, matrix metalloproteinase-9; NEDD, naphthyl ethylenediamine dihydrochloride; NIH, National Institutes of Health; NO, nitric oxide; Nrf2, nuclear factor erythroid 2-related factor 2; OD, optical density; PBS, phosphate-buffered saline; PFA, paraformaldehyde; PLD, Polydatin; PWL, paw withdrawal latency; RNS, Reactive nitrogen species; ROS, reactive oxygen species; SCI, Spinal cord injury; SEM, standard error of the mean.
